# A randomized controlled clinical trial of a Wim Hof Method intervention in women with high depressive symptoms

**DOI:** 10.1016/j.cpnec.2024.100272

**Published:** 2024-10-24

**Authors:** Robin Blades, Wendy Berry Mendes, Brian P. Don, Stefanie E. Mayer, Rebecca Dileo, Julia O'Bryan, Elena Fromer, Joanna Y. Guan, Sylvia S. Cheng, Ashley E. Mason, Aric A. Prather, Elissa S. Epel

**Affiliations:** a University of California, Los Angeles, Department of Psychology, USA; b Yale University, Department of Psychology, USA; c University of Auckland, School of Psychology, New Zealand; d University of California, San Francisco, Department of Psychiatry & Behavioral Sciences, USA; e University of Michigan, Department of Psychology, USA; f University of California, Davis, Center for Mind and Brain, USA; g University of California, Berkeley, Division of Epidemiology, USA

**Keywords:** Resilience, Hormesis, Depression, Stress, Breathwork, Paced breathing, Cortisol reactivity

## Abstract

**Objective:**

Stress is a driver of depression, and people with depression often struggle to cope with stress and anxiety. This study directly compares the mental health effects of a Wim Hof Method intervention to an active control condition (slow breathing) in women with high stress and high depressive symptoms.

**Methods:**

We randomized 84 healthy midlife women with high stress and high depressive symptoms to either: 1) the hormetic stress condition based on the Wim Hof Method (WHM) involving a breathing technique designed to induce intermittent hypoxia and cold showers (n = 41) or 2) an active comparison condition involving slow-paced breathing and warm showers (n = 43). We provided participants with daily audio instructions (15 min) for three weeks during the COVID-19 pandemic (2020–2021). Our primary outcomes were depressive symptoms, anxiety symptoms, and perceived stress collected at pre-intervention, post-intervention, and 3 months later. We also assessed daily stress rumination and affect with daily diary during the intervention, and participants completed a laboratory stressor, the Trier Social Stress Test, before and after the intervention, and provided samples for salivary cortisol reactivity.

**Results:**

Participants in the active control condition perceived the intervention to be more credible and expected greater mental wellbeing benefits compared to those in the Wim Hof Method condition. Differential attrition was observed with six participants (7 %) dropping out -- all from WHM condition. Among the participants who completed the intervention, both groups improved on mental health outcomes immediately after the intervention with a 24 % reduction in depressive symptoms, a 27 % reduction in anxiety symptoms, and 20 % reduction in perceived stress. Improvements were maintained at the 3-month follow-up with 46 % of the sample reporting mild or no depressive symptoms. Participants in the WHM condition had significant reductions in rumination after daily stressful events compared to those in the active control group. Both conditions had reduced daily negative affect across the intervention and lower peak cortisol reactivity to the lab stressor post-intervention.

**Conclusions:**

Counter to the preregistered predictions, and despite participants’ differing expectations, the interventions led to equivalent reductions in depressive symptoms, anxiety symptoms, and perceived stress, which were sustained at three months. They also produced comparable reductions in cortisol reactivity and daily negative affect. However, the WHM condition was associated with greater reduction in reported rumination after daily stressful events than the active control, a finding that needs replication with larger and more diverse samples.

## Highlights

• Comparison between a Wim Hof Method intervention vs. an active control condition in 84 women with high depressive symptoms.
• Both groups improved with a 24% reduction in depressive symptoms post-intervention.
• Improvements were maintained at the 3-month follow-up with 46 % of the sample reporting mild or no depressive symptoms.
• The Wim Hof Method group reported less daily rumination after stress compared to those in the active control post-intervention.
• Both conditions showed lower peak cortisol reactivity to the lab stressor post-intervention.

## 1 Introduction

Chronic stress can lead to increases in depression [1], which in turn enhances risk for morbidity and mortality [2]. During 2020 and 2021, over 300 million American adults had elevated depressive symptoms, driven largely by stress [3]. Daily stress causes persistent activation of the hypothalamic-pituitary-adrenal axis, which over time, can result in a dysregulated cortisol response and contribute to the development of depression [4]. Many studies have found relatively flat or blunted cortisol response curves in people with major depressive disorder [5,6], particularly in women [7], though findings are heterogeneous [8]. Low anticipatory cortisol is also associated with more stress-related depressive symptoms [9]. Normalization of HPA axis function appears to be critical for stable remission of depressive symptoms [10]. Easily accessible, diverse, and low-cost stress resilience interventions are needed to regulate the physiological stress response and treat depression. This study examines the psychophysiological effects of a Wim Hof Method intervention, which leverages intermittent hypoxia and cold showers to elicit a hormetic stress response in women with high stress and high depressive symptoms.

A promising new avenue of stress resilience research focuses on controlled exposure to a type of physiological arousal known as hormetic stress [11]. While chronic toxic stress can damage regulatory systems, repeated moderate-intensity acute stressors can produce positive psychological and physiological changes [12]. Hormetic stress may promote synaptic plasticity, which is disrupted in depression [13]. Training in hormetic stress may also normalize acute cortisol reactivity, in part through buffering against the effects of rumination [14]. Theoretically, hormetic stressors should produce cross-stressor adaption, in which exposure to one type of stressor fosters resilience to other future stressors [15].

The most well-studied hormetic stressor is exercise. It improves mood and reduces depressive symptoms, in part by promoting emotional recovery from the prolonged effects of stress [16]. Puterman et al. found that level of physical activity moderates the relationship between rumination and cortisol trajectory, such that sedentary participants with high rumination had more rapid increases, later peaks, and delayed recovery from stress compared to those with lower rumination [14]; active participants had equivalent cortisol trajectories, regardless of rumination. Exercise interventions may be able to reduce or normalize the stress reactivity in response to stress [17], though depressed participants may be more resistant to change [6].

While exercise has become an empirically supported treatment for depression [18], it is not accessible to those with lifestyle restrictions or physical disabilities. Additionally, low mood and stress are the most prevalent barriers to exercise [19]. It is thus important to test alternative hormetic stress interventions that are accessible to people with depression. Yet, there are few human studies that examine the effects of acute intermittent stress on depressive symptoms, beyond studies on exercise, leaving a wide gap in our translational knowledge.

The Wim Hof Method uses cycles of hyperventilation and breath-holding, as well as cold exposure, to induce hormetic stress. Preliminary evidence suggests that each of these components individually may be beneficial for depression. This breathing technique is intended to produce intermittent hypoxia, which theoretically conditions the HPA axis to respond more appropriately to mild stressors and sensitizes the negative glucocorticoid feedback system [20,21]. Though results are mixed [22], intermittent hypoxia is linked to improvements in depressive symptoms, both for animals [23] and humans [24,25]. It is being explored as potential treatment for depression, though these therapies are not accessible to the general public and require further testing [26]. Cold exposure activates the sympathetic nervous system [27], resulting in a physiological stress reaction similar to that induced by exercise. Preliminary evidence suggests that regular cold exposure decreases cortisol reactivity over the course of several weeks [28] and can reduce depressive symptoms in non-clinical samples [29].

While the Wim Hof Method is becoming increasingly popular among people seeking alternative health treatments, few scientific studies have investigated the efficacy of this method, and most research so far has focused on the immune system. The first case study of this method found that Wim Hof, the founder of the method, had a muted inflammatory response to an endotoxin injection compared to controls [30]. In the first group study, twelve men performed the Wim Hof Method for ten days and then were exposed to endotoxin injection [31]. They had lower pro-inflammatory responses, as well as fewer flu-like symptoms, compared to the control group. Another study found improvements in clinical disease markers in people with an autoimmune arthritic condition who practiced the Wim Hof Method [32].

Researchers are only just beginning to explore the potential mental health benefits of the Wim Hof Method. Early evidence suggests that intermittent hypoxia and cold exposure may have a unique synergy that is beneficial in reducing perceived stress -- beyond the effects of each hormetic stressor independently [33]. One small pilot study of 6 Antarctic expedition members found that an 8-week training program in Wim Hof Method breathing, cold exposures, and meditation significantly reduced self-reported stress and depressive symptoms compared to their control group, though they observed no differences in reduction of hair cortisol [34]. However, these benefits may not be as visible for individuals without significant stress and depressive symptoms, as one recent randomized controlled trial found no change in psychological (including perceived stress, positive affect, and negative affect) or physiological measures comparing healthy participants who completed a 15-day Wim Hof Method intervention and those assigned to a waitlist control group, both at rest or during a cold pressor test [35]. No studies we are aware of have investigated the effect of the Wim Hof Method on participants with high stress and depressive symptoms, in particular their acute stress response measured via salivary cortisol.

This study compares the mental health effects of a Wim Hof Method intervention to an active control condition involving slow breathing in women with high stress and high depressive symptoms (As Predicted registration #54051). We compared the efficacy of WHM to a low-arousal slow-paced breathing and warm shower comparison condition over a 3-week period. We assessed depressive symptoms, anxiety symptoms, and perceived stress at pre-intervention, post-intervention, and 3 months later. During the 21-day intervention, we collected daily diaries assessing stress rumination, positive affect, and negative affect. We also assessed salivary cortisol reactivity in response to a laboratory stressor, the Trier Social Stress Test, before and after the intervention. We preregistered that we expected participants in the WHM condition to experience reductions in depressive symptoms, anxiety symptoms, and perceived stress at post-intervention compared to the comparison group; that participants in the WHM condition would develop greater resilience to day-to-day stressors, demonstrated by decreases in daily stress rumination, decreases in negative affect, and increases in positive affect across the course of the intervention compared to those in the control condition; and given that acute stress responses measured with cortisol reactivity can be blunted or exaggerated with chronic stress and depression, we explored whether the different conditions would lead to changes in cortisol response profiles to the laboratory stressor.

## 2 Methods

*Transparency and Openness.* In this article, we report how we determined our data exclusions, analyses run, and all registered measures. We follow CONSORT guidelines. We include data, analysis code, and research materials on the Open Science Framework. Data were analyzed using SPSS version 28. The trial was pre-registered on As Predicted (#54051).

*Participants.* This study was approved by the UCSF Investigational Review Board (IRB# 18–25449) and participants signed informed consent for all procedures.

*Inclusion and exclusion criteria.* Due to known sex differences in stress responses, we recruited medically healthy women between the ages of 30 and 60 years old who reported moderate levels of depressive symptoms defined by a score more than or equal to 10 and less than 20 on the eight-item Patient Health Questionnaire (PHQ-8; [36]). The PHQ-8 is commonly used to screen for clinical depression. Participants with a score equal to or greater than 20, indicating severe major depression, were included only if they were concurrently in treatment (therapy or medication). (Although our inclusion criteria targeted moderately depressed women, depression measures collected after enrollment indicated that this sample had high depressive symptoms and was at risk for depression with an average CES-D score above 16.) We excluded individuals with self-reported chronic diseases, major psychiatric conditions (e.g., bipolar or schizophrenia), and/or severe anxiety defined by a score greater than or equal to 15 on the self-reported Generalized Anxiety Scale (GAD; [37]). We also excluded those with a self-reported BMI of more than 35, currently pregnant, smoking, or taking medications that affect the autonomic nervous system (beta blockers or ace inhibitors) or immune system. Additional safety exclusions included excluding those with physical disabilities or injuries that prohibit them from lying on the ground and individuals with Raynaud's disease that prohibits them from being randomized to cold-water exposure. Finally, we excluded individuals with panic disorder, as those who experience panic attacks on a regular basis will likely be afraid of hyperventilating and short-term breath control.

*Recruitment and screening.* We recruited participants (N = 84) through local community and organizational outreach, newsletters sent to University of California, San Francisco employees, flyers posted throughout the San Francisco Bay Area, online advertisements, published interviews, previous participant databases, and word of mouth, sharing our recruitment website https://www.stressresilience.net. The study took place between July 2020 and January 2021. We paid participants up to $470 for completion of all parts of the study, based on intervention and questionnaire completion. This payment included $150 for attending the pre-intervention lab visit, $100 for completing the 21-day intervention, $150 for attending the post-intervention lab visit, and $20 for completing the three-month follow-up questionnaire. We also provided incentive bonuses based on levels of daily intervention completion. Specifically, we provided $10 for having 51–65 % completion, $20 for having 66–84 % completion, and $40 for having 85–100 % completion. Additionally, we entered participants into a raffle to win a $200 Amazon gift card if they completed over 85 % of their intervention.

Interested participants completed a web-based self-report survey to determine the first phase of eligibility (i.e., based on age, sex, medical conditions). Study staff contacted potential participants who completed the web-based survey to conduct a phone interview to determine final eligibility. During the phone screening, study staff provided an overview of the study and confirmed eligibility criteria. They also requested that participants maintain the same level of medications and other daily routines (exercise/diet) throughout the study period and alert study staff of any changes. Once final eligibility was confirmed, prospective participants received the study consent form via DocuSign to provide their electronic signature and officially enroll in the study.

*Interventions.* We randomized participants to the two intervention conditions. Participants in all conditions received in-person, intervention-specific training after the first laboratory visit. Both interventions included detailed instructions for showering every morning. The breathing practices were designed to take roughly 15 min per session and required daily practice in the morning (or evening if their personal schedule did not allow). Participants also received a brief manual with FAQs (shared on OSF).

1) *Wim Hof Method (WHM) condition:* The WHM condition is based on the Wim Hof Method.^1^ This condition included rapid breathing and breath hold along with cold exposure in the form of cold showers. Wim Hof and other experts assisted in the development of the WHM condition, but to avoid any bias, they did not contribute beyond the development of the intervention. The WHM condition consisted of a breathing technique intended to induce hyperventilation followed by breath retention of one to 3 min (under the discretion of the participant). Once the participant determined they were not able to retain their breath any longer, they took a deep inhalation and held it for 15 s. Participants repeated this sequence for four cycles guided by an audio recording. Study staff reviewed safety information associated with this technique. The showering protocol included gradually exposing themselves to cold water by finishing their daily showers using cold water. They started with their normal shower routine at any temperature and transitioned to the coldest water option available at the end. The minimum duration of the cold shower slowly increased over the course of the intervention period: 30 s for days one to three, one to 3 min for days four to six, and two to 3 min for the remaining days. Total time duration not to exceed 3 min.
2) *Active control condition:* The low-arousal slow breathing and warm showers condition consisted of a 15-min breathing practice and a detailed description of showering instructions. The typical adult breathing rate is 12–18 breaths per minute, so we guided participants to breath at 8 breaths per minute. The audio led them to focus their attention on the inhalations and exhalations, and it was accompanied by rhythmic music to guide pace. We also instructed participants to extend their normal shower routine and transition to warm water for 3 min at the end (∼105 °F), which we estimated was within the typical range of shower temperature. We provided participants with thermometers to confirm the temperature setting. The warm showers were designed to match the cold showers in time and effort, but the temperature was not meaningfully different than one's normal shower and thus served as an active control shower activity.

### 2.1 Measures

*Trait Measures: Depressive symptoms, anxiety symptoms, and perceived stress.* We assessed depressive symptoms, anxiety symptoms, and perceived stress via online questionnaires prior to randomization to the intervention, after the 3-week intervention ended, and 3 months post-intervention. We measured depressive symptoms with the 20-item Center for Epidemiologic Studies Depression Scale (CES-D; [38]). Participants reported how frequently they have experienced depressive symptoms in the last week. The response options range from 0 (*rarely or none of the time*) to 3 (*most or all of the time*). In order to try to assess whether depression levels were more chronic or due to the pandemic, we also used a modified version of the 12-item Patient-Reported Outcomes Measurement Information System (PROMIS) for Depression at baseline to retrospectively assess depressive symptoms recalled before onset of the pandemic [39]. Participants recalled their depressive symptoms during a normal week in February 2020 (about a month before the COVID-19 outbreak) and reported if their experience has changed since then. The response options for the six pre-pandemic questions range from 1 (*never*) to 5 (*always*) with higher scores indicating more depressive symptoms before the COVID-19 outbreak. For each item, they also reported change in depressive symptoms since the COVID-19 outbreak from 1 (*it's gotten a lot worse*) to 5 (*it's gotten a lot better*) with higher responses indicating an improvement and lower responses indicating deterioration during the pandemic.

We assessed generalized anxiety symptoms with the 7-item GAD-7 [37], which assesses symptoms such as feeling nervous, restless, and worrying from 0 (not at all) to 4 (nearly every day). Scores above 10 indicate moderate anxiety [37]. We assessed perceived stress with the 10-item Perceived Stress Scale (PSS; [40]). Participants were asked to report how frequently they experienced stress in the last month. The response options range from 0 (*never*) to 4 (*very often*). Higher scores indicate more perceived stress.

*Daily Measures: stress rumination, positive affect, and negative affect.* We collected daily self-reports once a day in the evening for four days before participants began the intervention and throughout the 21-day intervention period. Each morning and evening of the 21-day intervention period, we sent a web link to participants’ preferred method of contact (email or SMS text) to complete a brief assessment. In these assessments, we measured stress rumination by asking participants to identify the most stressful event of the day and report “to what extent did you find yourself thinking about this stressful situation in the rest of the day afterward?” The response options range from 1 (*not at all*) to 5 (*a lot*) with higher scores indicating more rumination after stress. We measured positive and negative affect using a subset of four questions from the Modified Differential Emotions Scale (MDES; [41]). Each item asked participants how much they were feeling an affective state using three similar emotion terms. Two items assessed positive affect based on serenity (content, serene, peaceful) and happiness (glad, happy, joyful) and two items assessed negative affect based on stress (stressed, nervous, overwhelmed) and sadness (sad, downhearted, unhappy). The response options ranged from 1 (*not at all*) to 5 (*extremely*).

*Intervention expectations, credibility, and accessibility.* During the in-person intervention-specific training, we assessed participants’ expectations for how their intervention would impact their mental and physical health. We used two items similar to those used in Kox et al. [31] in which participants reported how much they expected their mental and physical health to improve on a scale from 1 to 10.

We also assessed the credibility and accessibility of the interventions immediately after the intervention via online questionnaire. The Credibility Expectancy Questionnaire (CEQ) has three questions related to how valuable the participants thought the intervention was [42]. The response options range from 1 to 9 with higher scores indicating more intervention credibility. The Treatment Acceptability Questionnaire (TAQ) has six questions to assess intervention acceptability [43]. The response options range from 1 to 7 with higher scores indicating more intervention acceptability.

*Cortisol reactivity during acute stress.* We collected salivary samples during two laboratory visits before and after the intervention. Three samples were collected per visit: a baseline sample before the Trier Social Stress Test (TSST), 20 min after the onset the TSST, and 40 min after onset of TSST (See Appendix A for more details on lab procedures.). Participants were provided with salivettes (Sarstedt, Nümbrecht, Germany) and instructed to chew on the cotton swab for 90 s. Samples were stored at a minimum of −20 °C. Then they were assayed to measure salivary free cortisol at the Salimetrics' SalivaLab (Carlsbad, CA) using the Salimetrics Salivary Cortisol Assay Kit (Cat. No. 1–3002). The assay had a lower limit of sensitivity of 0.007 μg/dL and a standard curve range from 0.012 to 3.0 μg/dL. The average intra-assay coefficient of variation was 4.60 %, and the inter-assay coefficient of variation was 6.00 %, which meets the manufacturers’ criteria for accuracy and repeatability, as well as the applicable NIH guidelines for Enhancing Reproducibility through Rigor and Transparency. The area under the curve with respect to ground (AUCg) and the area under the curve with respect to increase (AUCi) were calculated for each visit using methods established by Khoury et al. [44].

### 2.2 Analytic strategy

Data were cleaned and analyzed in SPSS 28. We adopted a statistical significance level of 0.05 for all statistical analyses. To examine change in participants' depressive symptoms, anxiety symptoms, and perceived stress, we conducted a series of linear mixed models that accounted for the nested nature of the data, as participants provided three reports over the course of 3 months (West et al., 2014). We examined the effect of time (baseline and post-intervention, as well as baseline and 3-month follow-up), the effect of condition (WHM vs. active control), and the interaction between time and condition. Each of these models were set with a random intercept and an unstructured covariance structure. Similarly, to examine change in participants’ cortisol reactivity during acute stress, measured as AUCi and AUCg, we examined the effect of time (baseline and post-intervention), the effect of condition (WHM vs. active control), and the interaction between time and condition.

To examine change in daily stress rumination, positive affect, and negative affect over the course of intervention, we conducted multilevel growth curve analyses (Bolger & Laurenceau, 2013). We specified all models with linear and quadratic effects for time to examine curvilinear patterns of change in stress rumination, positive emotions, and negative emotions during the 21-day intervention period. We included random intercepts and a random slope for the linear effect of time. We specified models with an unstructured covariance structure. As in the above analyses, we examined the pattern of change over the 21-day intervention period for those in the WHM condition, and then examined whether there was a significant difference between conditions. All 84 randomized participants were included in the analyses. No secondary “as-treated” analyses were performed due to the small difference in sample size.

## 3 Results

*Recruitment and enrollment.* Nine participants withdrew from the study *prior* to randomization due to family emergencies (n = 2), COVID-19 concerns (n = 2), concern over the study protocol (n = 2), and other time commitments (n = 3). As shown in Fig. S1 of study flow, 84 women were eligible, randomized, and consented and began the intervention. We used a random number generator to assign study condition in order to produce equivalent groups. Of the 84 participants who received intervention training, six dropped out during the 21-day intervention due to family emergencies (n = 2), COVID-19 concern (n = 1), study requirements (n = 1), and unknown reasons (n = 2). All six had been randomized to the WHM condition. Overall, 78 participants completed their assigned intervention (93 % retention). Eight of the 78 participants who completed the intervention (10.26 %) completed less than 70 % of the intervention. For the follow-up survey three months later, we invited only those who had high adherence (defined in our study registration as completing 70 % of intervention) for an ‘as treated’ analysis. We assessed daily intervention based on completion of the intervention survey, or if that was missing, on self-report in the evening diary. While 91 % of participants were eligible based on adherence, 65 % (n = 46) of eligible participants completed the 3-month follow-up survey (59 % of all completers). One of the 84 participants is missing self-report mental health data from baseline only and was included in models. See Appendix B for results of intervention expectations, credibility, and acceptability.

As reported in our demographic statistics (Table S1), the average age of the participants was 43.51 (SD = 10.39; 30 to 60) and the average BMI was 26 (SD = 4.79; range 19–37). The sample roughly reflected the demographics of the region, and participants on average were well-educated. The majority were unmarried (59.5 % unmarried; 39.3 % married; 1.2 % no answer). All participants were at least halftime employed or had a job in the past 12 months (including unpaid caretaking in the home). Despite randomization, there was a significant difference in race/ethnicity distribution between conditions (p = .042), with more Pacific Islander or Asian Americans in the WHM condition and more Black Americans in the active control condition. There were no other differences between the sociodemographic profiles. One participant had a score of 21 on the PHQ-8 questionnaire screening for depressive symptoms, and they were also already receiving concurrent treatment for depression, meeting our eligibility criteria. This individual was randomly assigned to the active control condition. An analysis of variance (ANOVA) also showed no significant differences in depressive symptoms, anxiety symptoms, or perceived stress at baseline between conditions.

### 3.1 Descriptive statistics and bivariate correlations

Consistent with the recruitment strategy, participants had moderate depressive symptoms on average at baseline screening (*M*_*PHQ-8*_ = 12.46; *SD* = 2.62) (though their pre-intervention scores using a different scale, the CES-D, indicated high levels of depressive symptoms). Only 33 % reported that their depressive symptoms worsened from pre-pandemic level, suggesting pre-existing depressive symptoms in two-thirds of participants. Seventeen of the 84 participants (20.24 % of the sample) were taking medication for mood (depression or anxiety) at baseline. Descriptive statistics and bivariate correlations for primary study variables are shown in Table S2.

As expected, trait-level distress measures were correlated with each other both before and after the intervention. At baseline, anxiety was negatively correlated with age, *r* (84) = −0.248, *p* < .023; at post-intervention, depressive symptoms, anxiety symptoms, and perceived stress were negatively correlated with age, *r* (78) = −0.273, *p* < .015; *r* (78) = −0.306, *p* < .006, and *r* (78) = −0.237, *p* < .036, respectively, suggesting that younger women suffered from more distress both before and after the intervention. However, age was not included in models predicting distress measures, because we do not have theoretical reason to believe that age should alter the impact of the intervention and we are not powered to examine age as a moderator. Given the lack of significant correlation, BMI was only included as a covariate in the cortisol models, where the literature has established a need to control for body mass [45].

### 3.2 Trait outcome 1: depression (CES-D)

**Fig. 1:**
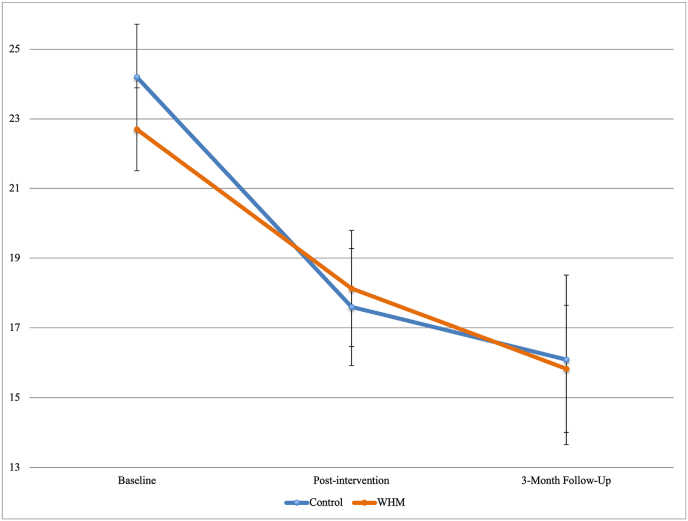
Depressive Symptoms (CES-D means and S.E.) by Condition at Each Wave of Data Collection.

Results of multilevel analyses examining change in depression found that, compared to baseline scores, participants in both conditions were less likely to report depressive symptoms post-intervention and at the 3-month follow-up (top section of Table 1), even after controlling for differential mental health treatment expectations (Table S4). The effect size of the reduction in depressive symptoms for those in both conditions was moderate (20.13 % reduction at post-intervention and 30.29 % reduction at the 3-month follow-up; Fig. 1). Of those from both groups who completed the 3-month follow up questionnaire, 46 % converted from high symptoms (16 or higher on CES-D at baseline, indicating moderate to severe clinical depression) to scores below 16 at post-intervention, indicating mild or no depression (Weissman et al., 1977).

**Table 1:** Results of multilevel analyses examining change in depressive symptoms, anxiety symptoms, and perceived stress before and after the intervention by condition with WHM condition as reference group.

Outcome	Predictor	B	p	95%CI		r
				*LL*	*UL*	
**CESD**	Intercept	22.656	<0.001	19.690	25.622	–
	Post-Intervention	−4.250	0.005	−7.197	−1.303	0.25
	3-Month Follow-Up	−5.762	0.002	−9.283	−2.242	0.27
	Condition	1.545	0.471	−2.685	5.776	0.06
	Post-Intervention x Condition	−2.355	0.255	−6.433	1.724	0.10
	3-month x Condition	−1.077	0.668	−6.033	3.880	0.04


**Outcome**	**Predictor**	**B**	***p***	**95%CI**		***r***
				***LL***	***UL***	
**GAD-7**	Intercept	8.605	<0.001	7.301	9.909	–
	Post-Intervention	−1.471	0.027	−2.776	−0.166	0.20
	3-Month Follow-Up	−2.800	<0.001	−4.357	−1.242	0.30
	Condition	0.172	0.856	−1.695	2.038	0.02
	Post-Intervention x Condition	−1.500	0.104	−3.314	0.313	0.15
	3-month x Condition	−0.453	0.685	−2.656	1.750	0.04


**Outcome**	**Predictor**	**B**	***p***	**95%CI**		***r***
				***LL***	***UL***	
**PSS**	Intercept	22.516	<0.001	20.850	24.181	–
	Post-Intervention	−3.700	<0.001	−5.394	−2.000	0.36
	3-Month Follow-Up	−5.217	<0.001	−7.244	−3.191	0.40
	Condition	−0.345	0.774	−2.720	2.030	0.02
	Post-Intervention x Condition	−1.401	0.241	−3.752	0.950	0.10
	3-month x Condition	0.088	0.952	−2.766	2.942	0.01

*Note. CI* = confidence interval; LL = lower limit; UL = upper limit. *r* was calculated using the method used by Kashdan and Steger (2006): r = √(t^2^/t^2^+df); CESD = Center for Epidemiologic Studies Depression Scale; GAD-7 = Generalized Anxiety Disorder scale; PSS = Perceived Stress Scale.

### 3.3 Trait outcome 2: anxiety (GAD-7)

As in the analysis of depression, participants in both conditions were less likely to report symptoms of anxiety symptoms at post intervention and at the 3-month follow-up (middle section of Table 1), even after controlling for differential mental health treatment expectations (Table S5). The effect size of the reduction in anxiety for both conditions was moderate (20.93 % reduction post-intervention and 39.30 % reduction at 3-months), and contrary to predictions there was no difference between conditions (Fig. S2).

### 3.4 Trait outcome 3: Perceived Stress Scale (PSS)

Participants in both conditions were less likely to report perceived stress at post intervention and at the 3-month follow-up (bottom section of Table 1), even after controlling for differential mental health treatment expectations (Table S6). The effect size of the reduction in perceived stress for both conditions was moderate, 17.26 % reduction at post-intervention and 24.83 % reduction at the 3-month follow-up, and again we did not observe any differences between conditions (Fig. S3).

In sum, we observed reductions in trait depressive symptoms, anxiety symptoms, and perceived stress over time at the end of the intervention and three months post-intervention. Contrary to expectations, the conditions did not differ from each other.

### 3.5 Daily outcome 1: stress rumination

**Fig. 2:**
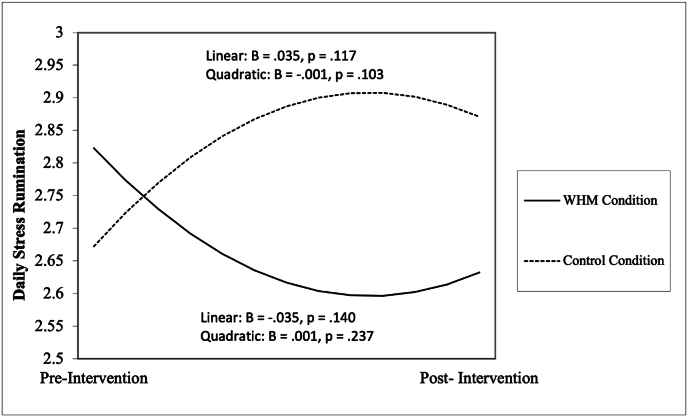
Quadratic growth trajectory of daily rumination after stress to explore quadratic time by condition effect (p = .048) for participants in the WHM condition compared to the active control condition.

We examined growth trajectories in participants’ self-reported rumination about the most stressful event of the day over the course of the 21-day intervention (top section of Table 2). There were no significant changes over time in either WHM or the active control. However, both the linear and quadratic interactions between condition and time were significant predictors of rumination after stress (p = .032 and p = .048, respectively) with small effect sizes (r = 0.05 for both). To decompose simple slopes of these interactions, we set either the active control or the Wim Hof Method intervention as the reference condition, and then examined the significance of the linear and quadratic coefficients. Participants in the active control condition demonstrated no significant linear or quadratic changes (Table S3.) Additionally, although the pattern of change for participants in the WHM condition was opposite to those in the active control condition (i.e., the trend was for a decrease in rumination which then leveled off later in the intervention), the linear or quadratic change components for rumination were also not significant for the those in the WHM (Fig. 2).

**Table 2:** Results of multilevel analyses examining change in daily stress rumination, positive affect, and negative affect across the intervention by condition with WHM condition as reference group.

Outcome	Predictor	B	*p*	95%CI		*r*
				*LL*	*UL*	
**Daily Stress** **Rumination**	Intercept	2.899	<0.001	2.574	3.224	–
	Time	−0.035	0.140	−0.081	0.011	0.04
	Time^2^	0.001	0.237	0.003	0.003	0.03
	Condition	−0.300	0.195	−0.756	0.155	0.09
	Time x Condition	0.070	0.032	0.006	0.133	0.05
	Time^2^ x Condition	−0.002	0.048	−0.005	−0.00003	0.05


**Outcome**	**Predictor**	**B**	***p***	**95%CI**		***r***
				***LL***	***UL***	
**Daily** **Positive Emotions**	Intercept	1.474	<0.001	1.218	1.729	–
	Time	0.011	0.474	−0.019	0.040	0.02
	Time^2^	−0.0005	0.388	−0.002	0.001	0.02
	Condition	0.189	0.300	−0.171	0.549	0.09
	Time x Condition	−0.016	0.430	−0.057	0.024	0.02
	Time^2^ x Condition	0.001	0.164	−0.0004	0.002	0.03


**Outcome**	**Predictor**	**B**	***p***	**95%CI**		***r***
				**LL**	**UL**	
**Daily** **Negative Emotions**	Intercept	1.263	<0.001	1.009	1.516	–
	Time	−0.055	<0.001	−0.084	−0.026	0.09
	Time^2^	0.002	<0.001	0.001	0.003	0.09
	Condition	−0.111	0.542	−0.468	0.247	0.05
	Time x Condition	0.025	0.220	−0.015	0.064	0.03
	Time^2^ x Condition	−0.001	0.098	−0.003	0.0002	0.04

*Note. CI* = confidence interval; LL = lower limit; UL = upper limit. *r* was calculated using the method used by Kashdan and Steger (2006): r = √(t^2^/t^2^+df).

### 3.6 Daily outcome 2: positive affect

We examined growth trajectories in participants’ self-reported positive affect over the course of the 21-day intervention (middle section of Table 2). There were no significant changes over time in either condition. Results suggest there were no changes in positive affect over time.

### 3.7 Daily outcome 3: negative affect

We examined growth trajectories in participants’ self-reported negative affect over the course of the 21-day intervention (bottom section of Table 2). We did not observe a main effect of condition, nor a condition by time interaction. There was a significant time effect with both linear and curvilinear changes in negative affect. Both groups showed a decline in daily negative affect soon after the start of the intervention, which then tapered off and increased again towards the end of the intervention. The effect sizes were small.

### 3.8 Cortisol reactivity

**Fig. 3:**
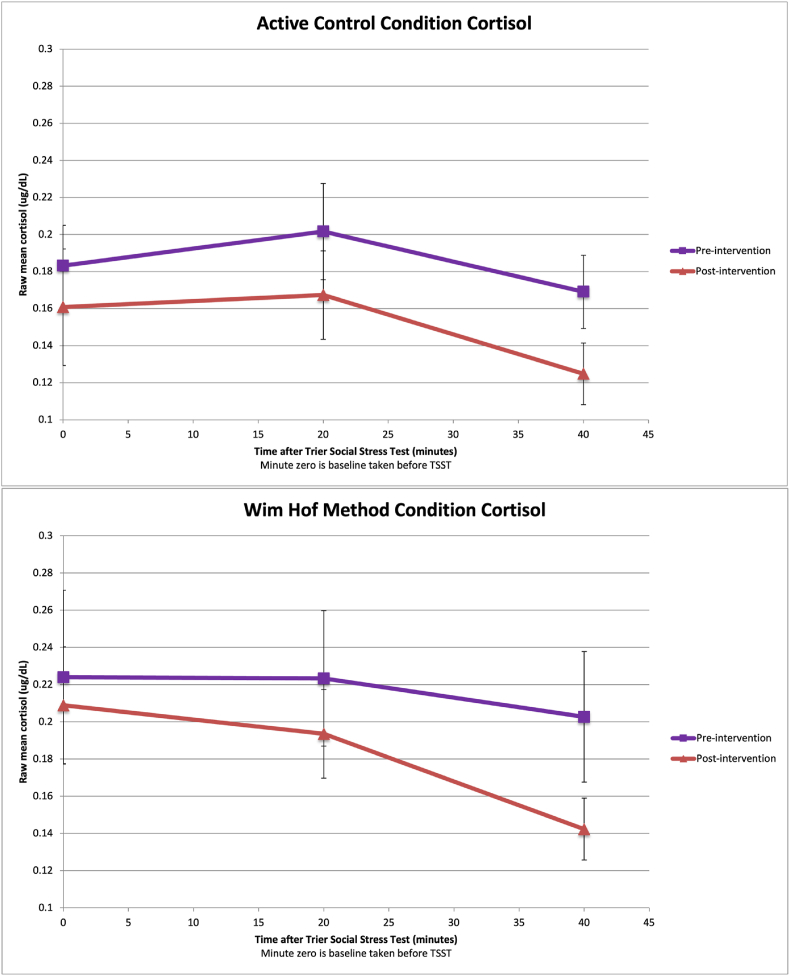
Raw Mean Salivary Cortisol Values of WHM and the Active Control Condition Groups Collected Before, 20 min after, and 40 min after before the Trier Social Stress Test Before and After the Intervention.

**Mean Cortisol levels.** In addition to examining AUC summary measures, we tested differences in cortisol at each point between groups. These comparisons demonstrated no differences pre-intervention (t(df) = 0.807(74), p = .422 at baseline; t(df) = 0.536(73), p = .593 at 20 min post-stressor; t(df) = 0.894(74), p = .374 at 40 min post-stressor) or post-intervention (t(df) = 0.1.388(74), p = .169 at baseline; t(df) = 0.900(72), p = .371 at 20 min post-stressor; t(df) = 0.929(72), p = .356 at 40 min post-stressor). However, an examination of within-group differences in cortisol comparing pre-intervention vs. post-intervention per group demonstrated a significant reduction in cortisol at 40 min post-stressor after the intervention compared to before the intervention for both groups (t(df) = 2.059(32), p = .024 in the WHM group; t(df) = 2.279(39), p = .028 in the active control group), as shown in Fig. 3.

**Total (AUCg) Cortisol**. Participants in the WHM and active control showed no significant change in cortisol between baseline and post-intervention, based on area under the curve with respect to ground (AUCg) (See the top section of Table 3.). Age and BMI were not significant predictors of AUCg.

**Table 3:** Results of multilevel analyses examining change in cortisol (AUCg and AUCi) across the intervention by condition with WHM condition as reference group.

Outcome	Predictor	B	*p*	95%CI		*r*
				*LL*	*UL*	
**AUCg**	Intercept	2.402	<0.001	1.627	3.176	–
	Post-Intervention	−0.038	0.730	−0.257	0.181	0.04
	Condition	−0.049	0.727	−0.329	0.230	0.03
	BMI	−0.009	0.486	−0.033	0.016	0.08
	Age	−0.007	0.252	−0.018	0.005	0.13
	Post-Intervention x Condition	−0.121	0.417	−0.418	0.175	0.10
**Outcome**	**Predictor**	**B**	***p***	**95%CI**		***r***
				***LL***	***UL***	
**AUCi**	Intercept	3.393	<0.001	3.154	3.632	–
	Post-Intervention	−0.169	<0.001	−0.261	−0.076	0.38
	Condition	0.020	0.675	−0.075	0.115	0.04
	BMI	−0.009	0.029	−0.016	−0.001	0.25
	Age	0.001	0.609	−0.003	0.004	0.06
	Post-Intervention x Condition	0.076	0.234	−0.050	0.201	0.14

*Note. CI* = confidence interval; LL = lower limit; UL = upper limit. *r* was calculated using the method used by Kashdan and Steger (2006): r = √(t^2^/t^2^+df); AUCg = area under the curve with respect to ground for cortisol; AUCi = area under the curve with respect to increase for cortisol.

**Reactive cortisol (AUCi).** Compared to their baseline scores, both conditions showed lower cortisol at post-intervention, based on area under the curve with respect to increase (AUCi) (See the bottom section of Table 3.). The effect size was moderate. Age was not a significant predictor of AUCi cortisol, though higher BMI predicted lower AUCi cortisol. These results suggest that participants in both conditions experienced a reduction in AUCi cortisol at post-intervention compared to baseline, controlling for age and BMI.

## 4 Discussion

Identifying effective, easily accessible, brief stress resilience interventions is critical to both prevent and reduce depression. This study assessed the impact of a high-arousal intermittent hypoxia and cold shower condition based on the Wim Hof Method compared to a low-arousal active control condition with slow-paced breathing and warm showers. Contrary to predictions, we did not observe differences between the conditions in changes in depressive symptoms, anxiety symptoms, or perceived stress. Instead, both conditions were associated with decreases in depressive symptoms (↓24 %), anxiety symptoms (↓27 %), and perceived stress (↓20 %) after three weeks of practice. These changes were sustained at the 3-month follow-up for depression (↓32 %), anxiety (↓39 %), and perceived stress (↓26 %) compared to baseline. Critically, the 3-month post-intervention comparison was a smaller sample – an “as treated” analysis (65 % of adherent participants, 59 % of all completers).

These findings are similar to our study with non-depressed, but highly stressed women comparing the same WHM intervention to exercise, meditation, and active control, where all groups improved similarly at three weeks [61]. This study suggests that stress resilience interventions targeting low and high arousal can similarly improve depressive symptoms, anxiety symptoms, and perceived stress even in a group with probable major depression. However, the improvements could also be a function of time given we did not include a standard no-treatment control group. Findings might be due to regression to the mean based on recruiting a moderately depressed and stressed sample.

There are several factors that could have contributed to a lack of differences across the two conditions. The active control condition led to greater mental health treatment expectations at baseline and higher perceived credibility than the WHM, which may have resulted in a more positive belief about the effect of the intervention throughout the three weeks. Medical studies have shown that depression is an outcome highly influenced by placebo effects [46]. Expectancy optimism has proven to be an important determinant of the physiological effects of the Wim Hof Method (such as immunological response to endotoxin) [47]. However, even after controlling for the differential mental health treatment expectations, participants in both groups had similar reductions in depressive symptoms, anxiety symptoms, and perceived stress, suggesting that the active control may have acted as a genuinely effective intervention.

There are two possible mechanisms through which the active control condition may have improved mental health in our participants. The slow-paced breathing audio closely guided participants to focus on their breath and promoted natural and comfortable breathing through the nose, matching the guidance in the WHM condition. This audio promoted mindful attention on the breath, which has demonstrated benefits for mental health [48]. Secondly, the rhythmic breathing was paced at 8 breaths per minute, which is slower than people's typical breathing rate and at a level that can benefit blood pressure [49]. Slow-paced breathing can also improve vagally-mediated heart rate variability and potentially improve stress management through afferent signaling to the brain [[50], [51], [52]]; improvements in heart rate variability may even strengthen neural networks related to emotion regulation [53]. Thus, our active control condition might have been effective at reducing stress by enhancing mindfulness and improving autonomic balance, beyond expectation effects. Indeed, in our examination of effects on the autonomic nervous system changes, the slow breathing active control condition appeared to have a beneficial effect [62]. The warm shower element of this condition is unlikely to be driving intervention effects, given that participants completed their warm shower as normal.

The only significant difference between the two interventions was daily rumination about a stressful daily event. Compared to participants in the active control condition, participants in the Wim Hof Method condition reported a reduction in rumination about a stressful daily event in the first two weeks of the intervention, though this effect faded by the end of the intervention. This finding suggests that WHM may reduce depressive symptoms through similar psychophysiological mechanisms as exercise, by promoting recovery from stress through reduced rumination [14]. One other study similarly found that the Wim Hof Method – but not breathwork or cold exposure alone – reduces perceived stress [33]. However, the rumination effects in our study were small, and this one difference would not survive a multi-comparison test.

Both groups showed reductions in daily negative affect, but no increases in positive affect. This is in contrast to our previous study on non-depressed women with high stress, where the WHM condition was associated with increases in daily positive affect (Epel et al., in review). Particularly for people under chronic stress, positive affect can protect against severe depression and is an important component of well-being [54]. We tentatively conclude that these low and high arousal stress resilience interventions do not appear effective for improving positive affect for people with depression, although this will require further study with larger samples.

Depression can blunt cortisol reactivity [5,6], in part due to elevated basal cortisol, whereas chronic stress can facilitate greater reactivity [55]. In the current sample recruited for high depressive symptoms, participants indeed appear to have a profile typical of depression with high basal cortisol and relatively flat reactivity curves; compared to a sociodemographically similar sample of women recruited for high perceived stress scores [61], this depressed sample had higher mean baseline cortisol at pre-intervention (M = 0.12, M = 0.20, respectively) and no increases above baseline. This cortisol trajectory could be due to anticipatory anxiety or chronically elevated baseline levels of cortisol. At post-intervention, the current sample showed reduced cortisol reactivity (based on area under the curve with respect to increase). Examination of time points revealed that women in both conditions had equally elevated baseline cortisol levels at each session, before and after the intervention, but a significant reduction in cortisol at 40 min post-stressor compared to pre-intervention, indicating improved recovery from acute stress in both groups. This finding could be explained by habituation and reduced novelty of the stressor, though the Trier Social Stress Test was modified for the second visit in an attempt to evoke the same physiological stress reaction. This improvement in recovery could also be driven in part to their lower levels of depressive symptoms, anxiety symptoms, and perceived stress.

Although the interventions were rated as equally acceptable, the WHM condition was less popular based on dropout, perceived as less likely to improve mental health, and perceived as less credible than the active control condition among participants with moderate depressive symptoms (see Appendix B for intervention expectations, credibility, and acceptability). While our previous study had similar dropout rates between all interventions, the six participants who dropped out of this study were all from the WHM. Interestingly, participants did not attribute their decision to leave the study to the characteristics of the intervention itself, but the uneven distribution suggests that they may have disliked the high-arousal breathing exercises and cold showers. Open-ended feedback indicated that participants wanted a better explanation for why they were doing the breathing exercises and cold showers. While the higher number of dropouts suggests that it may be more difficult to retain people with moderate depressive symptoms in the WHM condition, it may yet be a promising stress resilience intervention for people with depression.

*Limitations.* The design of this study is limited by its focus on distress measures, like depression, anxiety, and perceived stress, without a battery of well-being measures [56]. Also, the temperature for the cold exposure component of the Wim Hof Method intervention was not strictly controlled and likely varies based on geography, time of year, and participant adherence. A limitation to testing compliance is that we do not have measures of autonomic nervous system activity (RSA and PEP) during the home practices to test compliance and effects. However, at the end of the study, we asked participants to practice the breathing during a lab session when their autonomic activity was monitored. The results showed significant elevations in sympathetic activity for the Wim Hof Method breathing and parasympathetic activity for the slow breathing. This shows that the participants were able to create the expected autonomic states within each condition [62]. We did not assess use of intervention skills between the post-intervention and 3-month follow-up, so it remains unclear if the maintenance of mental health benefits in the subsample who continued to respond to questionnaires is due to the 3-week intervention solely or to continued practice.

Future studies would ideally add well-being questionnaires; measure water temperature or find other ways to standardize cold exposure; collect autonomic nervous system activity during home practices; and assess continued practice to interrogate sustained effects. While we requested that participants maintain a consistent daily routine in terms of diet and exercise, we also suggest that future studies track these routines throughout the study as they can impact mental and physical health outcomes. Larger sample sizes would facilitate detection of smaller effects and could assess the potential impact of anti-depressant treatments. Additionally, future studies should interrogate which components of the WHM intervention may be driving effects on mental health, or if they interact to create a greater cumulative effect. For example, a recent study tested the independent and combined effects of intermittent hypoxia and cold exposure on perceived stress, finding greater benefits related to synergy between these techniques [33]. Finally, we recommend that future studies include a non-active control group in addition to an active control group.

*Conclusion.* Contrary to preregistered predictions, we observed that both low- and high-arousal stress reduction interventions appeared effective for reducing depressive symptoms, anxiety symptoms, and perceived stress in people with moderate depressive symptoms. However, we cannot rule out the contribution of time effects, such that women recruited at moderate levels of depressive symptoms experienced reductions in symptoms over time due to regression to the mean. To our knowledge, this study is the first to establish the potential efficacy of a Wim Hof Method intervention with an intermittent hypoxia breathing technique and cold shower intervention compared to an active control condition with slow-paced breathing and warm showers in people with high depressive symptoms, with the limitation of not having a no-treatment control group. Notably, the outcomes with slow paced breathing at 8 breaths per minute were stronger than we expected, and participants reported liking the practices. We recommend further research into both fast-paced and slow-paced breathing exercises for patients with moderate to high depressive symptoms, as well as a close examination of the exact psychobiological pathways facilitating mental health benefits in order to optimize future intervention development.

## CRediT authorship contribution statement

**Robin Blades:** Writing – review & editing, Writing – original draft, Project administration, Investigation, Formal analysis, Data curation. **Wendy Berry Mendes:** Writing – review & editing, Supervision, Funding acquisition, Conceptualization. **Brian P. Don:** Writing – review & editing, Formal analysis. **Stefanie E. Mayer:** Writing – review & editing, Supervision, Conceptualization. **Rebecca Dileo:** Writing – review & editing, Project administration, Investigation, Data curation. **Julia O'Bryan:** Writing – review & editing, Project administration, Investigation, Data curation. **Elena Fromer:** Writing – review & editing, Project administration, Investigation, Data curation. **Joanna Y. Guan:** Writing – review & editing, Project administration, Investigation, Data curation. **Sylvia S. Cheng:** Writing – review & editing, Formal analysis, Data curation. **Ashley E. Mason:** Writing – review & editing, Conceptualization. **Aric A. Prather:** Writing – review & editing, Supervision, Funding acquisition, Conceptualization. **Elissa S. Epel:** Writing – review & editing, Writing – original draft, Supervision, Funding acquisition, Conceptualization.

## Compliance with ethical standards

The authors declare no conflict of interest. All procedures, including the informed consent process, were conducted in accordance with the ethical standards of the responsible committee on human experimentation (institutional and national) and with the Helsinki Declaration of 1975, as revised in 2000.

## Funding support

This study was supported by a grant from John W. Brick Mental Health Foundation, LSP Family Foundation, Joon Yun Family Foundation, NIA R24AG048024, and a National Academy of Medicine Catalyst Award.

## Declaration of competing interest

None

## Appendix A Lab Visit Procedures

*Pre-intervention lab visit.* Eligible participants were invited to the lab for their initial baseline visit. Autonomic nervous system measures were taken, described in a separate analysis (Don et al., 2023, in preparation). Participants sat quietly for 5 min after which the baseline salivary sample was collected. Participants then watched a neutral video, “The Appalachian Trail,” to create a passive rest period. Participants were asked to complete a Trier Social Stress Task (Kirschbaum et al., 1993). They were asked to present their qualifications for a dream job to a panel of two evaluators and perform a mental arithmetic task that involved counting backwards by sevens from 5-digit numbers. After a 5-min recovery period, the second salivary sample was collected (20 min after the start of the TSST). Then the third and final salivary sample was collected (40 min after the start of the TSST). Finally, participants were randomized and informed of their intervention condition. Participants watched an instructional intervention-specific video, then practiced their intervention in the lab, and discussed the daily diaries before heading home. Participants had a zoom video appointment with a trained coach later that day, or the next morning, to review questions about the intervention.

*Post-intervention lab visit.* After 21 days of intervention practice, participants returned to the lab for their follow-up visit. The follow-up was similar to the baseline visit with the exception of a modified TSST to avoid practice effects and ensure a stress response to a novel task. The speech task was more similar to the traditional TSST where the subject was instructed to give a 5-min speech on their strengths and weaknesses as a person. For the math task, participants were presented with a string of numbers of varying lengths that they were asked to recite backwards to the panel of evaluators by memory. At the end of the follow-up visit, participants were debriefed and paid for their time spent in the study.

## Appendix B Intervention Expectations, Acceptability, Credibility

At baseline, after the interventions were described to the participants, we examined whether differences existed between the conditions in expectations for improvements on mental and physical wellbeing. Results of an independent samples *t*-test demonstrated significant differences between the groups in terms of mental health expectations; t(df) = -2.663(75), p = .009. Those in the SPB condition expected greater improvements in their mental wellbeing (M = 7.30, SD = 1.43) than the FPB group did (M = 6.17, SD = 2.20). An independent samples *t*-test found no significant difference between the groups in terms of physical health expectations*;* t(df) = -0.904(75), p = .369.

Additional models were run to assess the impact of mental health treatment expectations on primary outcomes. We ran a three-way mixed model with depression as the outcome, and we found a significant interaction between group assignment and expectations. The effect size was small. We set the FPB group as the reference condition, and found that, in the SPB group, high mental health expectations significantly predict low depression overall (*B* = −2.484, 95 % CI [−4.545, −0.424], *p* = .019, *r* = 0.20), whereas in the FPB group they did not (*B* = 0.180, 95 % CI [−1.178, 1.539], *p* = .793, *r* = 0.02). The effect size was small. Mental health expectations and baseline depression were correlated at baseline in the SPB group (r = −0.374∗) and not the FPB group (r = 0.054), but there was no significant 3-way interaction between group assignment, expectations, and time. This finding suggests that expectations did not drive change in depression over the intervention, but rather initial depressive levels were associated with expectations (Table S4). We also assessed the impact of mental health treatment expectations on anxiety and perceived stress. We found that mental health treatment expectations did not predict anxiety or perceived stress, and there were no significant interactions between group assignment and expectations (Tables S5 and S6).

An independent samples *t*-test was performed to compare CEQ expectancy scores in the FPB and SPB conditions collected at post-intervention. There was a significant difference in CEQ credibility between the FPB (M = 16.68, SD = 7.43) and the SPB condition (M = 21.02, SD = 5.33); t(df) = -2.956(74), p = .004. This finding shows that participants experienced the SPB intervention as significantly more credible than the FPB intervention.

We performed an independent samples *t*-test to compare TAQ scores in the FPB and SPB condition collected at post-intervention. There was no significant difference in TAQ between the FPB (M = 34.75, SD = 5.55) and the SPB condition (M = 36.68, SD = 1.45); t(df) = -1.65(75), p = .104.

## Footnotes

1 Wim Hof Method is easily searchable on the internet, so we never referenced this method by name to prevent any expectations that might alter beliefs about the condition or subjective states.
