# Supplementary material for: A randomized controlled clinical trial of a Wim Hof Method intervention in women with high depressive symptoms
Source: Compr Psychoneuroendocrinol. 2024 Oct 24;20:100272. doi: 10.1016/j.cpnec.2024.100272 (PMC11599992; doi:10.1016/j.cpnec.2024.100272)
Supplement: Multimedia component 1 [file mmc1.docx]

**Supplementary Tables STARS-D**

**Table S1.**

*Demographics*

| **Demographic variable** | **STARS-D Sample** |
| --- | --- |
| Age; range, *M (SD)* | 30-60, 43.51 (10.39) |
| BMI; range, *M (SD)* | 19-37, 26.01 (4.79) |
| Marital Status; *N* (%)  Married  Not married  No answer | 33 (39.3%)  50 (59.5%)  1 (1.2%) |
| Education; *N* (%)  Associate’s Degree or fewer years of schooling  Bachelor’s Degree  Master’s Degree or more years of schooling | 4 (4.8%)  42 (50.0%)  38 (45.2%) |
| Race/ethnicity; *N* (%)  White  African American or Black  Asian or Pacific Islander  Latinx or Hispanic  Multiracial  Other | 45 (53.6%)  4 (4.8%)  24 (28.6%)  3 (3.6%)  7 (8.3%)  1 (1.2%) |
| PHQ-8; range, *M (SD)* | 10-21, 12.46 (2.62) |

*Note.* BMI = Body Mass Index; PHQ-8 = 8-Item Patient Health Questionnaire.

**Table S2.**

*Descriptive statistics and correlations*

| **Baseline** | **Mean (SD)** | **1** | **2** | **3** | **4** | **5** | **6** | **7** | **8** |
| --- | --- | --- | --- | --- | --- | --- | --- | --- | --- |
| 1. Age | 43.51 (10.39) | -- |  |  |  |  |  |  |  |
| 1. BMI | 26.03 (4.79) | .132 | -- |  |  |  |  |  |  |
| 1. Group^1^ | -- | -.039 | .176 | -- |  |  |  |  |  |
| 1. CESD | 23.44 (8.74) | -.098 | -.015 | .086 | -- |  |  |  |  |
| 1. GAD | 8.69 (4.29) | -.248* | -.105 | .020 | .663** |  |  |  |  |
| 1. PSS | 22.34 (4.66) | -.199 | -.013 | -.036 | .649** | .590** | -- |  |  |
| 1. AUCg | 1.86 (.650) | -.054 | -.170 | -.053 | .020 | .026 | -.106 | -- |  |
| 1. AUCi | 3.22 (.147) | -.026 | -.107 | .026 | -.003 | .028 | .023 | .337** | -- |
| **Post-intervention** | **Mean (SD)** | **1** | **2** | **3** | **4** | **5** | **6** | **7** | **8** |
| 1. Age | -- | -- |  |  |  |  |  |  |  |
| 1. BMI | 25.95 (4.89) | .113 | -- |  |  |  |  |  |  |
| 1. Group^1^ | -- | -.039 | .191 | -- |  |  |  |  |  |
| 1. CESD | 17.85 (10.42) | -.273* | .064 | -.026 | -- |  |  |  |  |
| 1. GAD | 6.28 (4.27) | -.306** | -.028 | -.117 | .778** |  |  |  |  |
| 1. PSS | 17.81 (6.22) | -.237* | .016 | -.125 | .826** | .745** | -- |  |  |
| 1. AUCg | 1.75 (.564) | -.199 | -.060 | -.177 | .124 | .077 | -.026 | -- |  |
| 1. AUCi | 3.09 (.255) | .040 | -.210 | .158 | -.121 | -.140 | -.076 | -.313** | -- |
|  |  |  |  |  |  |  |  |  |  |

*Note.* ^1^ 1 = WHM condition, 2 = Active Control condition; BMI = Body Mass Index; CESD = Center for Epidemiologic Studies Depression Scale; GAD = Generalized Anxiety Disorder; PSS = Perceived Stress Scale; AUCg = area under the curve with respect to ground; AUCi = area under the curve with respect to increase; **p* < .05 ***p* < .01.

**Table S3.**

*Results of Multilevel Analyses Examining Change in Daily Rumination After Stress Across the Intervention by Condition* *with Active Control Condition as Reference Group*

| **Outcome** | **Predictor** | **B** | ***p*** | **95%CI** | | ***r*** |
| --- | --- | --- | --- | --- | --- | --- |
|  |  |  |  | ***LL*** | ***UL*** |  |
| **Daily Stress** | Intercept | 2.599 | <.001 | 2.279 | 2.918 | - |
| **Rumination** | Time | 0.035 | 0.117 | -0.009 | 0.079 | 0.04 |
|  | Time^2^ | -0.001 | 0.103 | -0.003 | 0.0003 | 0.04 |
|  | Condition | 0.300 | 0.195 | -0.155 | 0.756 | 0.09 |
|  | Time x Condition | -0.070 | 0.032 | -0.133 | -0.006 | 0.05 |
|  | Time^2^ x Condition | 0.002 | 0.048 | 0.00003 | .005 | 0.05 |

*Note. CI* = confidence interval; LL = lower limit; UL = upper limit. *r* was calculated using the method used by Kashdan and Steger (2006): r = √(t^2^/t^2^+df)

**Table S4.**

*Results of Multilevel Analyses Examining Change in Depressive Symptoms Before and After the Intervention by Condition* *Controlling for Mental Health Expectations with WHM Condition as Reference Group*

| **Outcome** | **Predictor** | **B** | ***p*** | **95%CI** | | ***r*** |
| --- | --- | --- | --- | --- | --- | --- |
|  |  |  |  | ***LL*** | ***UL*** |  |
| **CESD** | Intercept | 23.161 | <.001 | 20.070 | 26.252 | - |
|  | Post-intervention | -4.911 | 0.003 | -8.150 | -1.672 | 0.28 |
|  | 3-month Follow-up | -5.951 | 0.003 | -9.876 | -2.026 | 0.27 |
|  | Condition | 1.588 | 0.471 | -2.756 | 5.931 | 0.06 |
|  | Mental health expectations | 0.180 | 0.793 | -1.178 | 1.539 | 0.02 |
|  | Post-Intervention x Condition | -1.942 | 0.393 | -6.431 | 2.547 | 0.08 |
|  | Post-Intervention x Expectations | -0.785 | 0.299 | -2.274 | 0.704 | 0.10 |
|  | Condition x Expectations | -2.664 | 0.035 | -5.132 | -0.196 | 0.18 |
|  | Post-Intervention x Condition x Expectations | 0.523 | 0.688 | -2.050 | 3.097 | 0.04 |
|  | 3-month x Condition | -1.070 | 0.723 | -7.029 | 4.889 | 0.03 |
|  | 3-month x Expectations | -1.012 | 0.223 | -2.646 | 0.623 | 0.11 |
|  | 3-month x Condition x Expectations | 0.838 | 0.631 | -2.608 | 4.283 | 0.04 |

*Note. CI* = confidence interval; LL = lower limit; UL = upper limit. *r* was calculated using the method used by Kashdan and Steger (2006): r = √(t^2^/t^2^+df); CESD = Center for Epidemiologic Studies Depression Scale; Mental health expectations are mean-centered.

**Table S5.**

*Results of Multilevel Analyses Examining Change in Anxiety Symptoms Before and After the Intervention by Condition* *Controlling for Mental Health Expectations with WHM Condition as Reference Group*

| **Outcome** | **Predictor** | **B** | ***p*** | **95%CI** | | ***r*** |
| --- | --- | --- | --- | --- | --- | --- |
|  |  |  |  | ***LL*** | ***UL*** |  |
| **GAD-7** | Intercept | 9.025 | <.001 | 7.623 | 10.427 | - |
|  | Post-intervention | -1.805 | 0.014 | -3.233 | -0.378 | 0.23 |
|  | 3-month Follow-up | -3.145 | <.001 | -4.877 | -1.413 | 0.32 |
|  | Condition | -0.201 | 0.84 | -2.171 | 1.768 | 0.02 |
|  | Mental health expectations | 0.114 | 0.716 | -0.502 | 0.73 | 0.03 |
|  | Post-Intervention x Condition | -1.102 | 0.272 | -3.079 | 0.876 | 0.11 |
|  | Post-Intervention x Expectations | -0.208 | 0.532 | -0.865 | 0.449 | 0.06 |
|  | Condition x Expectations | -0.535 | 0.346 | -1.654 | 0.584 | 0.08 |
|  | Post-Intervention x Condition x Expectations | -0.167 | 0.772 | -1.301 | 0.968 | 0.03 |
|  | 3-month x Condition | 0.194 | 0.884 | -2.437 | 2.825 | 0.01 |
|  | 3-month x Expectations | -0.172 | 0.638 | -0.893 | 0.549 | 0.04 |
|  | 3-month x Condition x Expectations | -0.274 | 0.722 | -1.795 | 1.248 | 0.03 |

*Note. CI* = confidence interval; LL = lower limit; UL = upper limit. *r* was calculated using the method used by Kashdan and Steger (2006): r = √(t^2^/t^2^+df); GAD-7 = Generalized Anxiety Disorder scale; Mental health expectations are mean-centered.

**Table S6.**

*Results of Multilevel Analyses Examining Change in Perceived Stress Before and After the Intervention by Condition Controlling for Mental Health Expectations with WHM Condition as Reference Group*

| **Outcome** | **Predictor** | **B** | ***p*** | **95%CI** | | | ***r*** |
| --- | --- | --- | --- | --- | --- | --- | --- |
|  |  |  |  | ***LL*** | | ***UL*** |  |
| **PSS** | Intercept | 22.404 | <.001 | 20.659 | 24.148 | | - |
|  | Post-intervention | -5.058 | <.001 | -6.823 | -3.293 | | 0.48 |
|  | 3-month Follow-up | -4.700 | <.001 | -7.249 | -2.152 | | 0.31 |
|  | Condition | 0.707 | 0.574 | -1.776 | 3.191 | | 0.05 |
|  | Mental health expectations | -0.737 | 0.218 | -1.915 | 0.441 | | 0.11 |
|  | Post-Intervention x Condition | 0.871 | 0.500 | -1.678 | 3.421 | | 0.06 |
|  | Post-Intervention x Expectations | -0.679 | 0.261 | -1.871 | 0.513 | | 0.11 |
|  | Condition x Expectations | 1.171 | 0.103 | -0.240 | 2.582 | | 0.14 |
|  | Post-Intervention x Control x Expectations | 0.225 | 0.761 | -1.237 | 1.687 | | 0.03 |
|  | 3-month x Condition | -1.180 | 0.492 | -4.567 | 2.207 | | 0.06 |
|  | 3-month x Expectations | -0.944 | 0.281 | -2.668 | 0.781 | | 0.10 |
|  | 3-month x Condition x Expectations | 0.230 | 0.816 | -1.728 | 2.189 | | 0.02 |

*Note. CI* = confidence interval; LL = lower limit; UL = upper limit. *r* was calculated using the method used by Kashdan and Steger (2006): r = √(t^2^/t^2^+df); PSS = Perceived Stress Scale; Mental health expectations are mean-centered.

**Figure S1.**

**Figure S2.**

*Anxiety Symptoms (GAD-7 means and S.E.) by Condition at Each Wave of Data Collection*

**
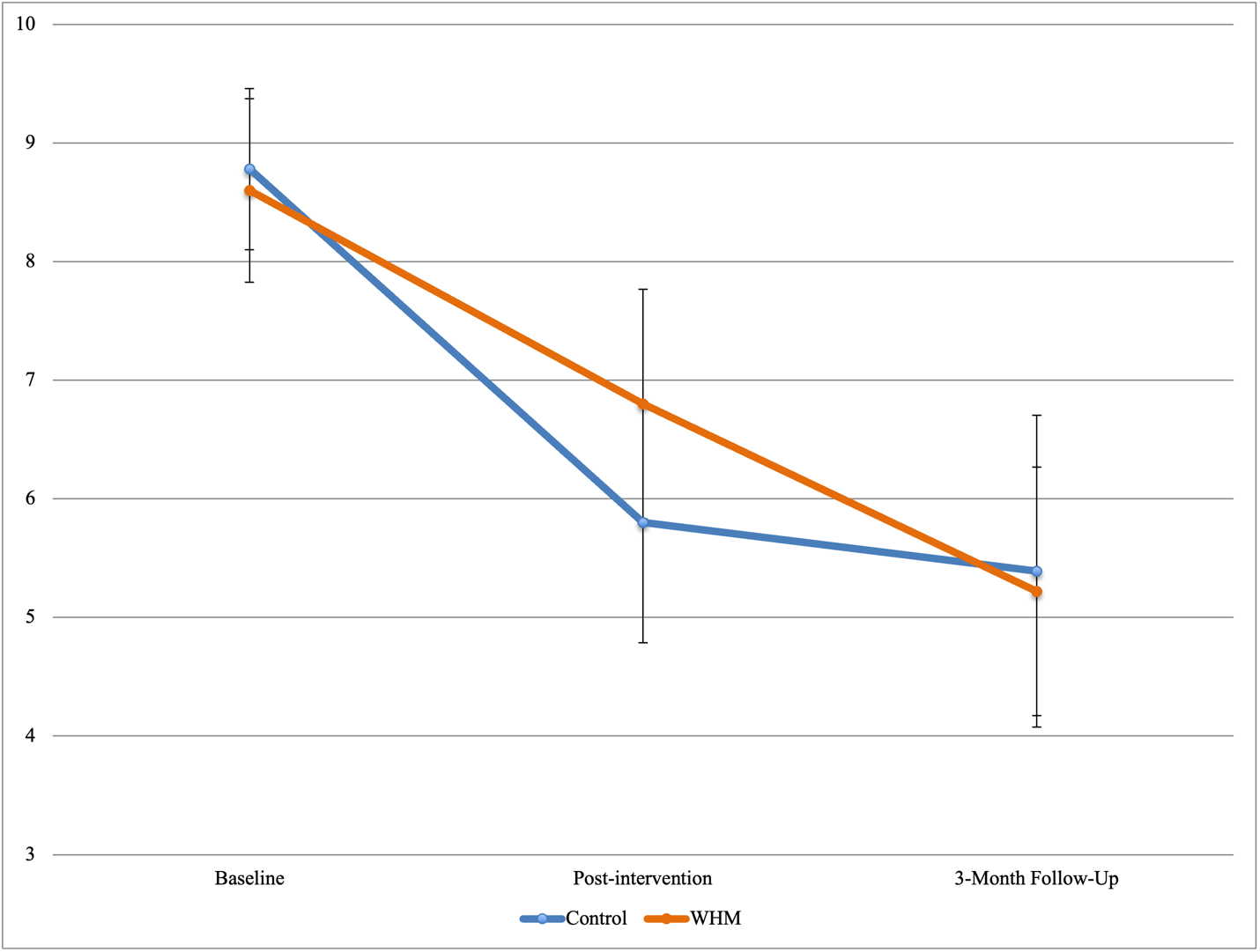
**

**Figure S3.**

*Perceived Stress (PSS means and S.E.) by Condition at Each Wave of Data Collection*

*
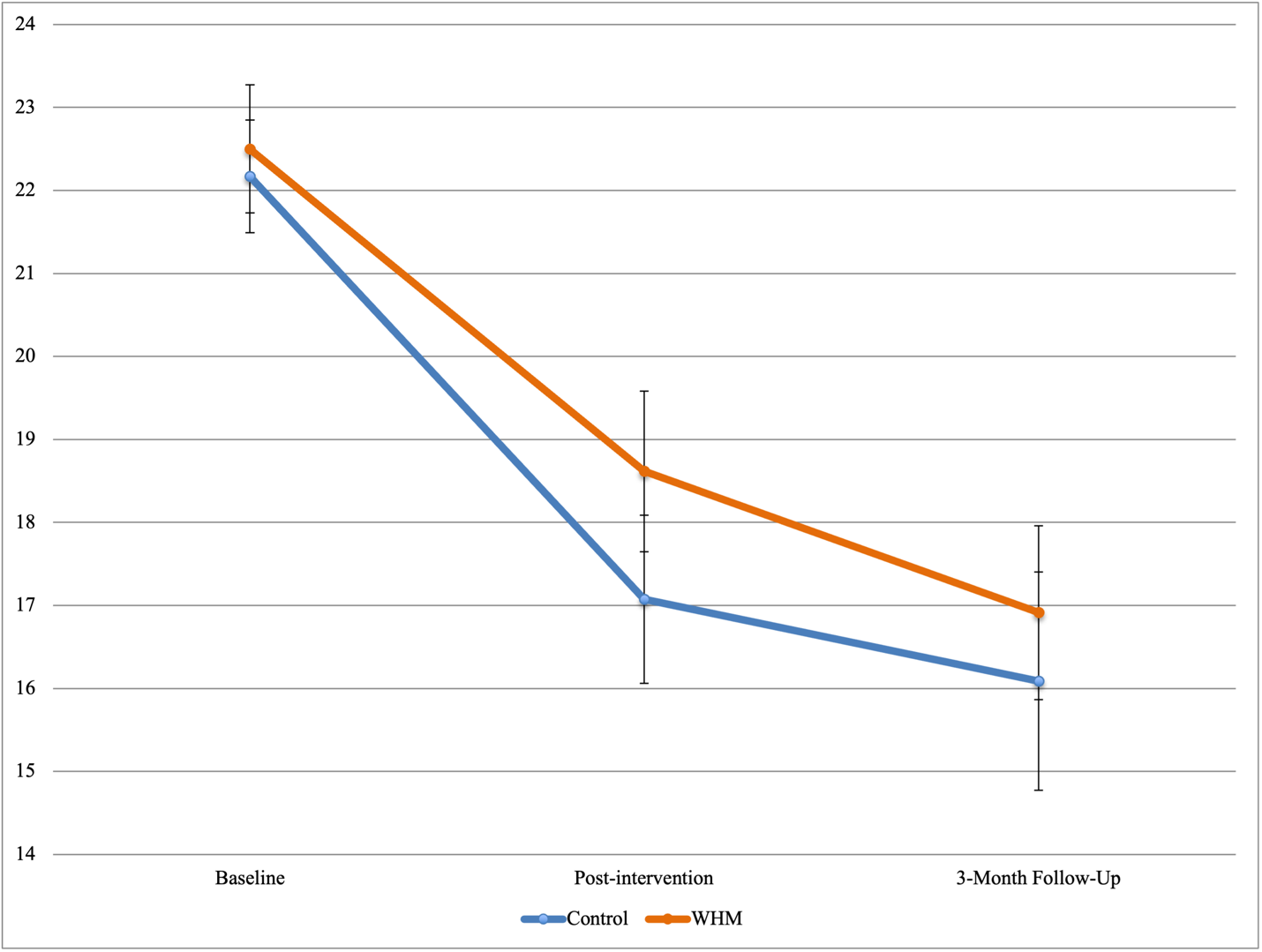
*
